# 2-Bromo-2-methyl-1-[4-(methyl­sulfan­yl)phen­yl]propan-1-one

**DOI:** 10.1107/S1600536812021472

**Published:** 2012-05-16

**Authors:** Fei Liu, Shi-Ying Ren, Wei-Ji Yang

**Affiliations:** a School of Life Science and Chemical Engineering, Huaiyin Institute of Technology, Huai’an, 223003 Jiangsu Province, People’s Republic of China; b Graduate School, Zhejiang Chinese Medical University, Hangzhou, 310053, People’s Republic of China

## Abstract

In the title compound, C_11_H_13_BrOS, the thio­ether unit and the phenyl ring adopt an essentially planar conformation, with a maximum deviation of 0.063 Å. In the crystal, mol­ecules are linked by C—H⋯O hydrogen bonds, extending in zigzag chains along the *b* axis. A weak intra­molecular C—H⋯Br hydrogen bond is also observed, which forms an *S*(6) ring motif.

## Related literature

For general background to the properties of the title compound, a key inter­mediate for the preparation of a UV initiator, and its synthesis, see: Zhao *et al.* (2010 ▶); Liu *et al.* (2010 ▶). For related structures, see: Anuradha *et al.* (2008 ▶); Moreno-Fuquen *et al.* (2011 ▶).
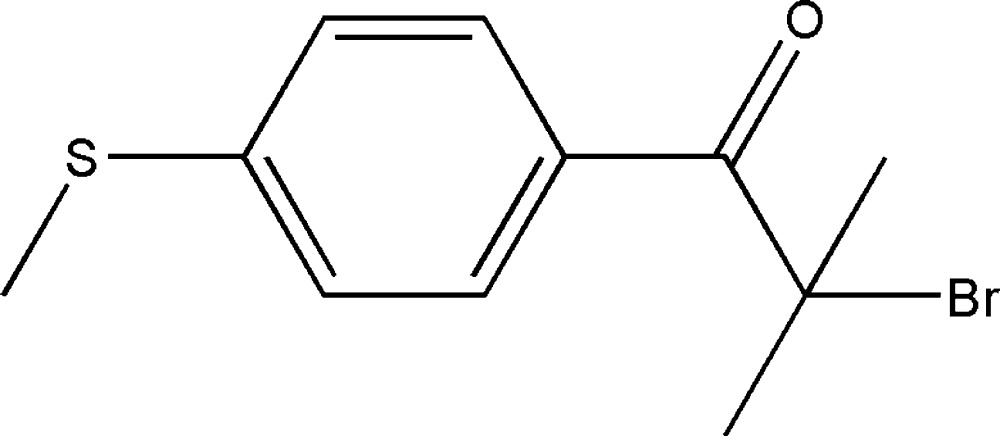


## Experimental

### 

#### Crystal data

C_11_H_13_BrOS
*M* *_r_* = 273.18
Monoclinic, 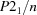

*a* = 11.061 (3) Å
*b* = 7.120 (2) Å
*c* = 14.721 (4) Å
β = 97.638 (3)°
*V* = 1149.1 (5) Å^3^
*Z* = 4
Mo *K*α radiation
μ = 3.73 mm^−1^
*T* = 153 K
0.27 × 0.23 × 0.18 mm

#### Data collection

Rigaku AFC10/Saturn724+ diffractometer
Absorption correction: multi-scan (*ABSCOR*; Higashi, 1995 ▶) *T*_min_ = 0.433, *T*_max_ = 0.551
9900 measured reflections
3625 independent reflections
2901 reflections with *I* > 2σ(*I*)
*R*_int_ = 0.033

#### Refinement

*R*[*F* ^2^ > 2σ(*F* ^2^)] = 0.032
*wR*(*F* ^2^) = 0.073
*S* = 1.00
3625 reflections
130 parameters
H-atom parameters constrained
Δρ_max_ = 0.57 e Å^−3^
Δρ_min_ = −0.72 e Å^−3^

### 

Data collection: *CrystalClear* (Rigaku/MSC, 2008 ▶); cell refinement: *CrystalClear*; data reduction: *CrystalClear*; program(s) used to solve structure: *SHELXS97* (Sheldrick, 2008 ▶); program(s) used to refine structure: *SHELXL97* (Sheldrick, 2008 ▶); molecular graphics: *SHELXTL* (Sheldrick, 2008 ▶); software used to prepare material for publication: *publCIF* (Westrip, 2010 ▶) and *PLATON* (Spek, 2009 ▶).

## Supplementary Material

Crystal structure: contains datablock(s) I, global. DOI: 10.1107/S1600536812021472/bg2458sup1.cif

Structure factors: contains datablock(s) I. DOI: 10.1107/S1600536812021472/bg2458Isup2.hkl

Supplementary material file. DOI: 10.1107/S1600536812021472/bg2458Isup3.cml

Additional supplementary materials:  crystallographic information; 3D view; checkCIF report

## Figures and Tables

**Table 1:** Hydrogen-bond geometry (Å, °)

*D*—H⋯*A*	*D*—H	H⋯*A*	*D*⋯*A*	*D*—H⋯*A*
C11—H11*A*⋯O1^i^	0.98	2.47	3.359 (2)	150
C5—H5⋯Br1	0.95	2.78	3.387 (2)	123

Symmetry code: (i) 
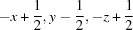
.

**Fig. 1.**
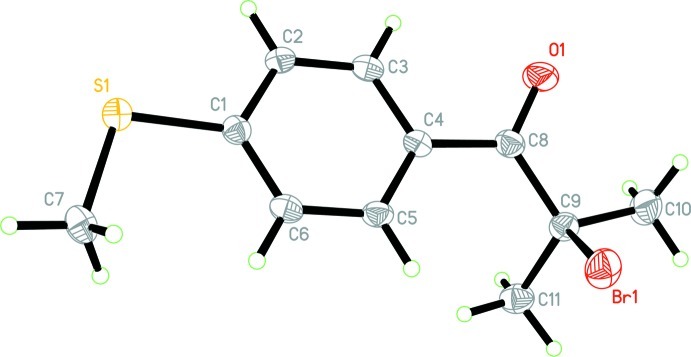
The molecular structure of (I), showing the atom-numbering scheme and displacement ellipsoids drawn at the 50% probability level.

**Fig. 2.**
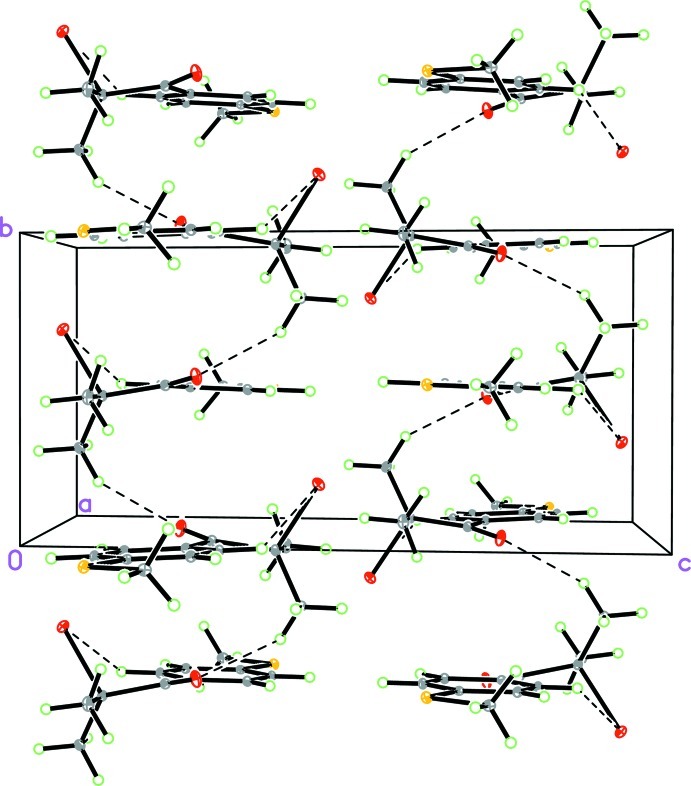
The packing of the crystal structure of (I), showing the intramolecular hydrogen bonds and the zigzag chains formed by C—H···O hydrogen bonds (dotted lines).

## supplementary crystallographic information

### Comment

The title compound is a key intermediate for the preparation of 2-methyl-1-[4-(methylthio)phenyl]-2-(4-morpholinyl)-propanone, which is used as a UV initiator (Zhao *et al.*, 2010). It was prepared from thioanisole, which was reacted with isobutyryl chloride in the presence of aluminium cloride, followed by bromination with bromine/acetic acid (Liu *et al.*, 2010).

C—S bond lengths and the C—S—C angle agree with those in (*E*)-3-(4-fluorophenyl)-1-[4-(methylsulfanyl)phenyl]prop-2-en-1-one (Anuradha *et al.*, 2008). The S—C*sp*^3^ bond length (1.7954 Å) is longer than the S—C*sp*^2^ one (1.7548 Å) and the C—Br distance length (1.9962 Å) is in the normal range for this type of bonds (Moreno-Fuquen *et al.*, 2011).

The thioether moiety and phenyl ring adopt an essentially planar conformation with a maximum deviation of 0.063 Å (Fig. 1). In the crystal, molecules are linked by C—H···O hydrogen bonds, extending as zigzag chains along the *b* axis (Fig. 2). In addition, a weak intramolecular C—H···Br hydrogen bond is also observed, forming an *S*(6) ring motif. This H-bond geometry is listed in Table 1.

### Experimental

To a mixture of dichloroethane (50 ml) and aluminium cloride (17.4 g, 130 mmol) was added isobutyryl chloride (13.8 g, 130 mmol) at 298 K. Thioanisole (10.8 g, 100 mmol) was added dropwise to the mixture. After completion, it was poured into diluted hydrochloric acid and the organic layer was extracted.

To the organic layer, acetic acid (7.4 g, 123 mmol) and 30% hydrogen peroxide solution (7.4 g, 65 mmol) were added. Then, bromine (10.4 g, 65 mmol) was added at 303 K. After completion, water was added, and the organic layer was washed by 5% sodium bicarbonate solution and concentrated to yield the title compound with a yield of 77.3%. The crude product was recrystallized by slow evaporation from ethanol to give the single crystals used for data collection.

### Refinement

C—bound H atoms were positioned geometrically with C—H 0.95 Å and 0.98 Å for C*sp*^2^ and methyl C, respectively, and were treated as riding on their parent atoms, with *U*_iso_(H)=1.2 *U*_eq_(C).

### Crystal data

**Table:**

C_11_H_13_BrOS	*F*(000) = 552
*M**_r_* = 273.18	*D*_x_ = 1.579 Mg m^−^^3^
Monoclinic, *P*2_1_/*n*	Mo *K*α radiation, λ = 0.71073 Å
Hall symbol: -P 2yn	Cell parameters from 4327 reflections
*a* = 11.061 (3) Å	θ = 2.2–31.0°
*b* = 7.120 (2) Å	µ = 3.73 mm^−^^1^
*c* = 14.721 (4) Å	*T* = 153 K
β = 97.638 (3)°	Block, colourless
*V* = 1149.1 (5) Å^3^	0.27 × 0.23 × 0.18 mm
*Z* = 4	

### Data collection

**Table:**

Rigaku AFC10/Saturn724+ diffractometer	3625 independent reflections
Radiation source: Rotating Anode	2901 reflections with *I* > 2σ(*I*)
Graphite monochromator	*R*_int_ = 0.033
Detector resolution: 28.5714 pixels mm^-1^	θ_max_ = 31.0°, θ_min_ = 2.8°
phi and ω scans	*h* = −16→14
Absorption correction: multi-scan (*ABSCOR*; Higashi, 1995)	*k* = −10→8
*T*_min_ = 0.433, *T*_max_ = 0.551	*l* = −21→21
9900 measured reflections	

### Refinement

**Table:**

Refinement on *F*^2^	Primary atom site location: structure-invariant direct methods
Least-squares matrix: full	Secondary atom site location: difference Fourier map
*R*[*F*^2^ > 2σ(*F*^2^)] = 0.032	Hydrogen site location: inferred from neighbouring sites
*wR*(*F*^2^) = 0.073	H-atom parameters constrained
*S* = 1.00	*w* = 1/[σ^2^(*F*_o_^2^) + (0.0339*P*)^2^] where *P* = (*F*_o_^2^ + 2*F*_c_^2^)/3
3625 reflections	(Δ/σ)_max_ = 0.001
130 parameters	Δρ_max_ = 0.57 e Å^−^^3^
0 restraints	Δρ_min_ = −0.72 e Å^−^^3^

### Special details

**Table:**

Geometry. All e.s.d.'s (except the e.s.d. in the dihedral angle between two l.s. planes) are estimated using the full covariance matrix. The cell e.s.d.'s are taken into account individually in the estimation of e.s.d.'s in distances, angles and torsion angles; correlations between e.s.d.'s in cell parameters are only used when they are defined by crystal symmetry. An approximate (isotropic) treatment of cell e.s.d.'s is used for estimating e.s.d.'s involving l.s. planes.
Refinement. Refinement of *F*^2^ against ALL reflections. The weighted *R*-factor *wR* and goodness of fit *S* are based on *F*^2^, conventional *R*-factors *R* are based on *F*, with *F* set to zero for negative *F*^2^. The threshold expression of *F*^2^ > σ(*F*^2^) is used only for calculating *R*-factors(gt) *etc*. and is not relevant to the choice of reflections for refinement. *R*-factors based on *F*^2^ are statistically about twice as large as those based on *F*, and *R*- factors based on ALL data will be even larger.

### Fractional atomic coordinates and isotropic or equivalent isotropic displacement parameters (Å^2^)

**Table:**

	*x*	*y*	*z*	*U*_iso_*/*U*_eq_	
Br1	0.327247 (17)	0.69311 (3)	0.037381 (13)	0.03062 (7)	
S1	0.86419 (4)	0.48317 (6)	0.36691 (3)	0.02352 (10)	
O1	0.25965 (11)	0.5378 (2)	0.26208 (9)	0.0358 (3)	
C1	0.71222 (15)	0.4889 (2)	0.31441 (12)	0.0184 (3)	
C2	0.62070 (15)	0.4850 (2)	0.37198 (12)	0.0211 (3)	
H2	0.6426	0.4798	0.4366	0.025*	
C3	0.49965 (15)	0.4886 (2)	0.33555 (12)	0.0213 (3)	
H3	0.4389	0.4856	0.3755	0.026*	
C4	0.46416 (15)	0.4968 (2)	0.24059 (11)	0.0186 (3)	
C5	0.55563 (15)	0.5004 (2)	0.18392 (12)	0.0201 (3)	
H5	0.5339	0.5061	0.1193	0.024*	
C6	0.67786 (16)	0.4957 (2)	0.22041 (12)	0.0212 (3)	
H6	0.7387	0.4972	0.1805	0.025*	
C7	0.94956 (16)	0.4878 (3)	0.27138 (13)	0.0256 (4)	
H7A	0.9245	0.3824	0.2303	0.031*	
H7B	1.0368	0.4773	0.2937	0.031*	
H7C	0.9340	0.6063	0.2380	0.031*	
C8	0.33064 (16)	0.5063 (2)	0.20746 (12)	0.0214 (4)	
C9	0.27806 (15)	0.4717 (2)	0.10720 (12)	0.0213 (4)	
C10	0.13996 (16)	0.4720 (3)	0.09560 (14)	0.0325 (4)	
H10A	0.1084	0.4581	0.0305	0.039*	
H10B	0.1110	0.5908	0.1185	0.039*	
H10C	0.1111	0.3673	0.1303	0.039*	
C11	0.32472 (17)	0.2949 (2)	0.06566 (13)	0.0264 (4)	
H11A	0.3003	0.1846	0.0987	0.032*	
H11B	0.4139	0.2998	0.0705	0.032*	
H11C	0.2900	0.2861	0.0010	0.032*	

### Atomic displacement parameters (Å^2^)

**Table:**

	*U*^11^	*U*^22^	*U*^33^	*U*^12^	*U*^13^	*U*^23^
Br1	0.03163 (12)	0.03130 (13)	0.02925 (11)	0.00122 (8)	0.00520 (8)	0.00951 (8)
S1	0.0197 (2)	0.0277 (2)	0.0229 (2)	0.00113 (17)	0.00177 (16)	−0.00186 (17)
O1	0.0213 (7)	0.0622 (10)	0.0251 (7)	0.0015 (7)	0.0078 (5)	−0.0100 (7)
C1	0.0189 (8)	0.0163 (8)	0.0201 (8)	0.0003 (6)	0.0028 (6)	−0.0008 (6)
C2	0.0234 (9)	0.0247 (9)	0.0155 (8)	0.0015 (7)	0.0036 (6)	−0.0007 (6)
C3	0.0230 (8)	0.0229 (9)	0.0194 (8)	0.0001 (7)	0.0077 (6)	−0.0014 (7)
C4	0.0193 (8)	0.0178 (8)	0.0193 (8)	0.0004 (6)	0.0054 (6)	−0.0005 (6)
C5	0.0213 (8)	0.0240 (9)	0.0158 (8)	−0.0008 (7)	0.0060 (6)	−0.0001 (6)
C6	0.0218 (8)	0.0230 (9)	0.0204 (8)	−0.0011 (7)	0.0085 (6)	0.0001 (7)
C7	0.0187 (8)	0.0266 (10)	0.0323 (10)	−0.0008 (7)	0.0063 (7)	0.0006 (8)
C8	0.0207 (8)	0.0234 (9)	0.0209 (8)	0.0004 (7)	0.0058 (6)	−0.0006 (7)
C9	0.0199 (8)	0.0253 (9)	0.0192 (8)	−0.0021 (7)	0.0047 (6)	0.0019 (7)
C10	0.0189 (9)	0.0487 (13)	0.0294 (10)	−0.0018 (8)	0.0018 (7)	0.0004 (9)
C11	0.0278 (10)	0.0273 (11)	0.0244 (9)	−0.0048 (7)	0.0045 (7)	−0.0039 (7)

### Geometric parameters (Å, º)

**Table:**

Br1—C9	1.9962 (17)	C6—H6	0.9500
S1—C1	1.7548 (17)	C7—H7A	0.9800
S1—C7	1.7954 (19)	C7—H7B	0.9800
O1—C8	1.217 (2)	C7—H7C	0.9800
C1—C6	1.386 (2)	C8—C9	1.533 (2)
C1—C2	1.404 (2)	C9—C10	1.514 (2)
C2—C3	1.375 (2)	C9—C11	1.520 (2)
C2—H2	0.9500	C10—H10A	0.9800
C3—C4	1.402 (2)	C10—H10B	0.9800
C3—H3	0.9500	C10—H10C	0.9800
C4—C5	1.395 (2)	C11—H11A	0.9800
C4—C8	1.494 (2)	C11—H11B	0.9800
C5—C6	1.387 (2)	C11—H11C	0.9800
C5—H5	0.9500		
			
C1—S1—C7	103.15 (9)	H7A—C7—H7C	109.5
C6—C1—C2	118.63 (16)	H7B—C7—H7C	109.5
C6—C1—S1	124.05 (13)	O1—C8—C4	119.29 (16)
C2—C1—S1	117.32 (13)	O1—C8—C9	118.05 (16)
C3—C2—C1	120.47 (16)	C4—C8—C9	122.63 (14)
C3—C2—H2	119.8	C10—C9—C11	110.31 (15)
C1—C2—H2	119.8	C10—C9—C8	110.88 (14)
C2—C3—C4	121.25 (15)	C11—C9—C8	114.50 (15)
C2—C3—H3	119.4	C10—C9—Br1	106.31 (12)
C4—C3—H3	119.4	C11—C9—Br1	108.46 (12)
C5—C4—C3	117.90 (16)	C8—C9—Br1	105.95 (11)
C5—C4—C8	124.62 (16)	C9—C10—H10A	109.5
C3—C4—C8	117.45 (14)	C9—C10—H10B	109.5
C6—C5—C4	121.01 (16)	H10A—C10—H10B	109.5
C6—C5—H5	119.5	C9—C10—H10C	109.5
C4—C5—H5	119.5	H10A—C10—H10C	109.5
C1—C6—C5	120.74 (15)	H10B—C10—H10C	109.5
C1—C6—H6	119.6	C9—C11—H11A	109.5
C5—C6—H6	119.6	C9—C11—H11B	109.5
S1—C7—H7A	109.5	H11A—C11—H11B	109.5
S1—C7—H7B	109.5	C9—C11—H11C	109.5
H7A—C7—H7B	109.5	H11A—C11—H11C	109.5
S1—C7—H7C	109.5	H11B—C11—H11C	109.5
			
C7—S1—C1—C6	−0.14 (17)	C4—C5—C6—C1	−0.5 (3)
C7—S1—C1—C2	179.45 (13)	C5—C4—C8—O1	−166.18 (16)
C6—C1—C2—C3	−0.2 (3)	C3—C4—C8—O1	12.1 (2)
S1—C1—C2—C3	−179.84 (13)	C5—C4—C8—C9	15.8 (3)
C1—C2—C3—C4	−0.2 (3)	C3—C4—C8—C9	−165.99 (15)
C2—C3—C4—C5	0.2 (3)	O1—C8—C9—C10	−3.6 (2)
C2—C3—C4—C8	−178.13 (15)	C4—C8—C9—C10	174.51 (16)
C3—C4—C5—C6	0.1 (2)	O1—C8—C9—C11	−129.15 (17)
C8—C4—C5—C6	178.36 (16)	C4—C8—C9—C11	48.9 (2)
C2—C1—C6—C5	0.6 (3)	O1—C8—C9—Br1	111.37 (16)
S1—C1—C6—C5	−179.83 (13)	C4—C8—C9—Br1	−70.55 (18)

### Hydrogen-bond geometry (Å, º)

**Table:**

*D*—H···*A*	*D*—H	H···*A*	*D*···*A*	*D*—H···*A*
C11—H11*A*···O1^i^	0.98	2.47	3.359 (2)	150
C5—H5···Br1	0.95	2.78	3.387 (2)	123

Symmetry code: (i) −*x*+1/2, *y*−1/2, −*z*+1/2.
